# A Case of Lymphomatoid Granulomatosis Presenting as a Cervical Mass

**DOI:** 10.7759/cureus.23534

**Published:** 2022-03-27

**Authors:** Masafumi Yoshida, Ayumi Mori, Kai Kanemoto, Shizuka Shoji, Asayo Furukawa

**Affiliations:** 1 Department of Otolaryngology, Showa General Hospital, Tokyo, JPN; 2 Department of Otolaryngology and Head and Neck Surgery, The University of Tokyo Hospital, Tokyo, JPN

**Keywords:** lymphomatoid granulomatosis, incisional biopsy, horner's sign, differential diagnosis, cervical mass

## Abstract

An 89-year-old man presented with the chief complaint of a sore throat and a mass in the right side of his neck that tended to increase in size. He displayed the right Horner's sign, and imaging findings showed a 65-mm mass in the right side of the neck, invading the carotid artery. There were no other obvious lesions. The differential diagnosis was carcinoma of an unknown primary or neurogenic tumor. An incisional biopsy was performed, and the pathological diagnosis was lymphomatoid granulomatosis. The patient was started on oral prednisolone, but the disease progressed, and his general condition worsened. Therefore, supportive care was provided. This was a very rare case of a cervical lesion of lymphomatoid granulomatosis.

## Introduction

Many diseases that present with cervical masses, such as inflammatory diseases and both benign and malignant neoplastic lesions. To differentiate these, pathological diagnosis by biopsy may be necessary. In this article, we report a case of lymphomatoid granulomatosis that was initially suspected to be a malignant tumor but was diagnosed by biopsy as lymphomatoid granulomatosis, which rarely presents as a cervical cancer lesion.

## Case presentation

An 89-year-old man was referred to our hospital for a thorough examination after a computed tomography (CT) scan revealed a tumor-like lesion in the right side of his neck. At the time of the initial examination, the swelling was observed from the right submandibular region to the upper neck, and submucosal swelling was observed on the right wall of the oropharynx. However, no obvious neoplastic lesions were observed in the oral cavity or pharynx. Right eyelid drooping and pupil contraction were considered to represent Horner’s syndrome. There was no evidence of laryngeal paralysis, but he experienced severe dysphagia due to swelling of the pharynx, weight loss, and decreased mobility.

**Figure 1:**
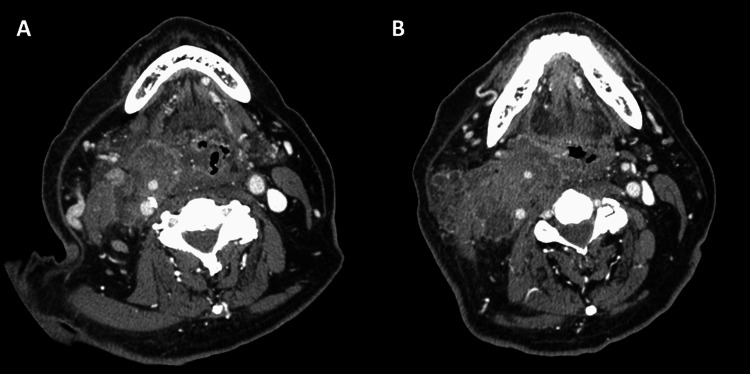
Contrast-enhanced CT of the neck. (A) and (B) Contrast-enhanced CT: a 65-mm-long bifurcated mass was found mainly in the right carotid artery space. The margins are irregular, and the contrast effect is heterogeneous. The right common carotid artery bifurcation to the internal and external carotid arteries is involved in the mass. CT: computed tomography.

A neck-to-pelvis contrast CT showed a 65-mm-long bifurcated mass mainly in the carotid artery space, inferior to the right parapharyngeal space. The right common carotid artery bifurcation to the internal and external carotid arteries was entrapped by the tumor, which was enlarged compared to a previous CT scan obtained one month earlier. There were no obvious pathological findings except in the neck region (Figure 1).

**Figure 2:**
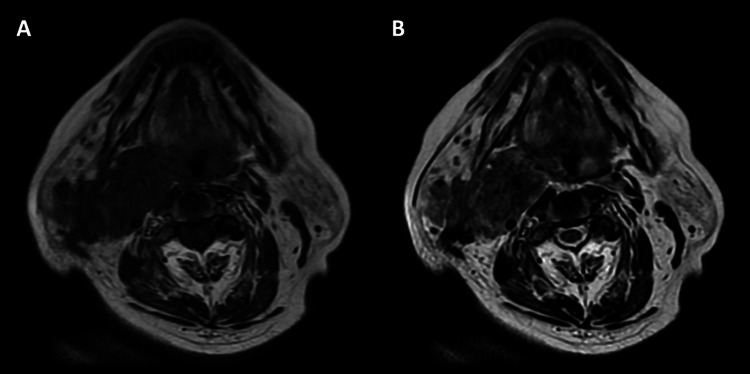
MRI of the neck. (A) and (B): Plain MRI: soft dense mass with indistinct borders in the right side of the neck, showing an intermediate signal on T1-weighted images and a heterogeneous low-to-moderate signal on T2-weighted images. MRI: magnetic resonance imaging.

Plain magnetic resonance imaging (MRI) of the neck showed an ill-defined soft tissue mass in the right side of the neck with an intermediate signal on T1-weighted images and a heterogeneously low-to-moderate signal on T2-weighted images (Figure 2).

Diffusion-weighted imaging showed a high signal with a low apparent diffusion coefficient was low. The imaging findings, rapid tendency to increase in size, and Horner's sign strongly suggested the possibility of malignancy, including parotid carcinoma with cervical lymph node metastasis, carcinoma of unknown primary or neurogenic tumor, such as malignant peripheral schwannoma, based on the findings of enlargement between the internal and external carotid arteries, primarily in the carotid artery space. Fine needle aspiration cytology was performed twice, but it was difficult to distinguish between benign and malignant tumors because of the high degree of destruction and degeneration. Therefore, an incisional biopsy was planned.

**Figure 3:**
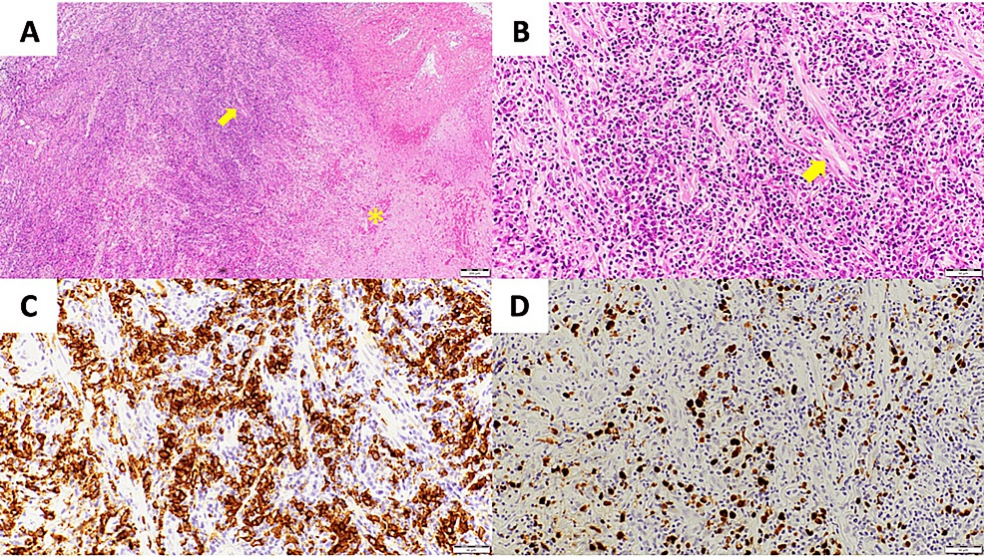
Pathological findings of the biopsy specimen. (A) Hematoxylin and eosin (HE) staining (40×): large round cells with atypical nuclei are seen perivascularly with extensive geographic necrosis (*). An arrow signifies a blood vessel. (B) HE staining (200×): perivascular infiltration of atypical cells and destruction of blood vessels are observed. An arrow signifies a blood vessel. (C) CD20 immunostaining (200×): the atypical cells seen in (a) and (b) are diffusely CD20 positive. (D) Epstein-Barr virus (EBV)-encoded RNA-in situ hybridization (EBER-ISH) (200×): the atypical cells seen in (a) and (b) are predominantly positive on EBER-ISH.

A biopsy of the tumor under local anesthesia revealed numerous large round cells with enlarged atypical nuclei in a perivascular lesion with an image of vascular disruption. These atypical cells were diffusely positive for CD20 (a marker of B cells), and many were positive on Epstein-Barr virus (EBV)-encoded RNA-in situ hybridization (EBER-ISH). Some cells were also positive for PAX-5 (a transcription factor expressed throughout B cell maturation) and CD30 (a marker of activated B cells and T cells), negative for CD15 and anaplastic lymphoma kinase (ALK), and had a relatively large number of CD3-positive small T cells. Based on these findings, the patient was diagnosed with lymphomatoid granulomatosis (Figure 3). 

A large number of EBER-ISH-positive cells (200-250 cells/high-power field (HPF)) and extensive necrosis were found, and a diagnosis of grade 3 was made. However, after a review by the hematology department, it was determined that the patient's age and general condition did not warrant intense chemotherapy, and oral prednisolone was started at 35 mg per day. Although mild improvement of neck swelling was observed for about one week after the start of treatment, the effect was not long-lasting, and three weeks after the start of treatment, the patient was unable to take oral medication and was admitted to another hospital for palliative care.

## Discussion

Lymphomatoid granulomatosis is a rare B-cell lymphoproliferative disease caused by the Epstein‒Barr virus [1-3]. It is most common in patients in their 40s to 60s, with a male-to-female ratio of approximately 2:1. In most cases, the lung is the main site of the disease. Histologically, various small-to-large lymphoid cells with nuclear atypia invade blood vessels, forming angiogenic or vasculopathy lesions. In addition to the lungs, the skin, central nervous system, liver, and kidneys are also affected [4]. It has been classified according to the ratio of large B cells to the number of EBER-ISH-positive cells [2,4,5]. This case corresponded to grade 3, which was the most malignant grade. Although it is a rare disease and there are few reports on its prognosis, the median survival is reported as 14‒72 months, and the prognosis is poor, particularly in cases of grade 2‒3 cases and those with central nervous system involvement. There is no established treatment, but in grade 2‒3 patients, combination therapy with low-dose oral cyclophosphamide and prednisone or chemotherapy with multiple drugs, including rituximab, an anti-CD20 antibody, is often used [4,6].

In this case, there were no lesions other than those in the neck region, which has not been reported in any case previously. Although the pathogenesis and prognosis of cervical lesions are unknown, the rapid increase in size and worsening of the patient’s general condition in the month before diagnosis and the lack of response to steroids suggest that the disease was aggressive. Initially, cancer and neurogenic tumors were suspected, and lymphoproliferative diseases such as these were not considered in the differential diagnosis. Normally, incisional biopsy is not recommended for cancer metastases due to the risk of dissemination. However, in cases such as this patient, where the diagnosis cannot be narrowed down by imaging or cytology, biopsy should be considered promptly even though there is a high possibility of malignancy and even if the tumor is unresectable, and radiotherapy or chemotherapy is the main treatment option, to confirm the diagnosis and determine the appropriate treatment plan.

## Conclusions

We experienced a very rare case of lymphomatoid granulomatosis with neck involvement. Initially, cancer metastasis was suspected, but an incisional biopsy confirmed the diagnosis. Due to his advanced age and rapid progression of the disease, the patient had to be transferred to best supportive care (BSC) early after treatment. Through the accumulation of cases in the future, we await the establishment of diagnostic and therapeutic methods for this disease.
